# Gene flow drives genomic diversity in Asian Pikas distributed along the core and range‐edge habitats in the Himalayas

**DOI:** 10.1002/ece3.10129

**Published:** 2023-05-24

**Authors:** Nishma Dahal, Melia G. Romine, Sunita Khatiwara, Uma Ramakrishnan, Sangeet Lamichhaney

**Affiliations:** ^1^ Biotechnology Division CSIR‐Institute of Himalayan Bioresource Technology Palampur Himachal Pradesh India; ^2^ National Centre for Biological Sciences, TIFR Bangalore India; ^3^ School of Biomedical Sciences Kent State University Kent Ohio USA; ^4^ Forest and Environment Department, Government of Sikkim Gangtok India; ^5^ Department of Biological Sciences Kent State University Kent USA

**Keywords:** gene flow, genetic diversity, Himalayas, pika

## Abstract

Studying the genetic variation among different species distributed across their core and range‐edge habitats can provide valuable insights into how genetic variation changes across the species' distribution range. This information can be important for understanding local adaptation, as well as for conservation and management efforts. In this study, we have carried out genomic characterization of six species of Asian Pikas distributed along their core and range‐edge habitats in the Himalayas. We utilized a population genomics approach using ~28,000 genome‐wide SNP markers obtained from restriction‐site associated DNA sequencing. We identified low nucleotide diversity and high inbreeding coefficients in all six species across their core and range‐edge habitats. We also identified evidence of gene flow among genetically diverse species. Our results provide evidence of reduced genetic diversity in Asian pikas distributed across the Himalayas and the neighboring regions and indicate that recurrent gene flow is possibly a key mechanism for maintaining genetic diversity and adaptive potential in these pikas. However, full‐scale genomics studies that utilize whole‐genome sequencing approaches will be needed to quantify the direction and timing of gene flow and functional changes associated with introgressed regions in the genome. Our results represent an important step toward understanding the patterns and consequences of gene flow in species, sampled at the least studied, yet climatically vulnerable part of their habitat that can be further used to inform conservation strategies that promote connectivity and gene flow between populations.

## Graphical abstract

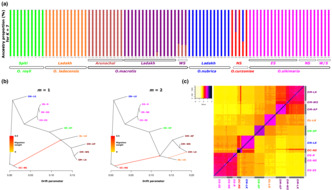

## 1 INTRODUCTION

The populations of a species at the outer limits of their geographic distribution beyond which they cannot survive or thrive are defined as range‐edge populations (Angert et al., 2020). Species face unique challenges at their range edge that can impact their survival and reproduction and may ultimately affect the long‐term viability of the population. These challenges can include changes in climate, habitat fragmentation, reduced genetic diversity, and increased competition or predation (Angert et al., 2020). As a result, range‐edge populations may have different characteristics than populations at the center of the distribution range, such as smaller population sizes, lower genetic diversity, and local adaptations. Several factors can impact species at its range‐edge including (a) climatic conditions, e.g., changes in climatic conditions at the range‐edge can limit the species' ability to survive and reproduce, (b) physical barriers such as mountains, rivers, and oceans can limit the dispersal of species and create a range‐edge, (c) availability of suitable habitat, (d) alteration in community composition leading to competition or predation. Ultimately, genetic factors such as reduced genetic diversity, inbreeding, and genetic drift can impact the fitness of individuals and the viability of range‐edge populations. The unique challenges faced by the range‐edge populations are often the focus of conservation efforts and understanding the dynamics of range‐edge populations has been important for the conservation and management of species, particularly in the face of environmental change (Rehm et al., 2015).

The impact of the environment on the population dynamics of a species varies across its geographical range (Smith et al., 2019), hence the study of the populations across their geographic range is key for studying the climate sensitivity of the species. Such studies can help to identify genetically distinct populations and inform conservation efforts aimed at preserving genetic diversity (Razgour et al., 2013). Additionally, genetic characterization can provide insights into the adaptive potential of the species and their ability to respond to environmental change (Razgour et al., 2019). Low genetic diversity as a response to reductions in effective population size towards the range edge is a widely accepted concept in the field of conservation biology (Eckert et al., 2008; Vucetich & Waite, 2003). This reduction in genetic variation may cause populations in range‐edge to have low adaptive potential and an increased risk of extinction (Pierce et al., 2017). However, gene flow from nearby populations can introduce new genetic variation into the range‐edge population, potentially increasing adaptive potential and reducing the risk of extinction (Pfennig et al., 2016).

**FIGURE A1:**
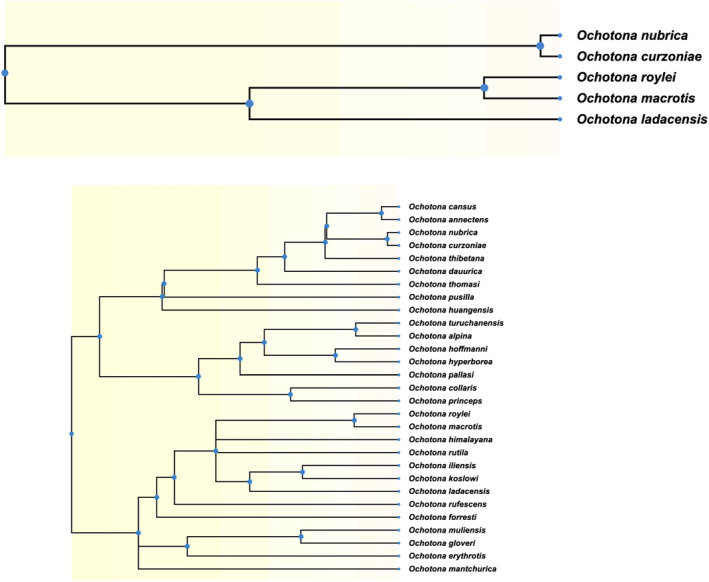
Expected phylogenetic tree using all species we currently used in this study. O. sikimaria is not currently present in the Timetree database (above). Expected phylogenetic tree of all 29 Ochotona species currently present in the Timetree database (below).

Pikas are small, herbivorous mammals that belong to the *Ochotonidae* family. It belongs to only one extant genus *Ochotona* with 33 species spread across the Asian highlands, easternmost Europe, and North America (Lissovsky, 2014; Lissovsky et al., 2017, 2019, 2022; Figure A1). Pikas are sensitive to changes in temperature and precipitation (Rinnan & Lawler, 2019). The evolutionary history of extant pikas supports their climate sensitivity as the ancestral, warm‐temperature‐loving lineage is known to have gone extinct (Ge et al., 2023; Wang et al., 2020). Their habitats in high‐altitude environments are typically narrow and isolated in the Himalayas (Dahal et al., 2020).

Despite the pika being one of the most abundant species in the high‐altitude regions of Himalaya and Qinghai Tibetan Plateau (QTP), studies on Asian pikas are limited. Of the six Asian pikas species reported from the Himalayas (Dahal et al., 2020), the ecology and genetics of only two species—*O. curzoniae* and *O. roylei* have been explored (Bhattacharyya et al., 2013, 2014, 2019; Bhattacharyya & Ishtiaq, 2019; Liu et al., 2022; Qu et al., 2017), but see the recent study by Ge et al. (2023). Previous studies on Asian pikas are mostly restricted to resolving taxonomic ambiguities and reports of distinct lineages (Dahal et al., 2017; Koju et al., 2017; Lissovsky et al., 2017, 2019, 2022). Interspecies hybridization and introgression within subgenus *Ochotona* have been proposed (Lissovsky et al., 2019, 2022). Population genetic studies in Asian pikas are however limited (Bhattacharyya & Ishtiaq, 2019; Ge et al., 2023; Zhang et al., 2017) and significantly lower than vast literature on studies of population dynamics and evolution in North American Pikas, e.g., (Holtz et al., 2016; Klingler et al., 2021; Robson et al., 2016; Waterhouse et al., 2017; Westover et al., 2020). A recent study by Ge et al. (2023) examined six closely related species of Asian pikas and reported gene flow among species within subgenus *Ochotona*, at their contact zone in the Himalayas. The Himalayas and the Qinghai Tibetan Plateau (QTP) form major distribution centers for at least two species‐rich subgenera (*Conothoa* and *Ochotona*) out of a total of five recognized subgenera of pikas (Lissovsky et al., 2022; Wang et al., 2020). Recently diverged species groups in the Himalayas reportedly have parapatric geographic ranges. For example, *O. sikimaria* and *O. nubrica* show elevation segregation with *O. curzoniae*, while *O. nubrica* and *O. sikimaria* are segregated based on latitude (Dahal et al., 2020). Deeply diverged sister groups *O. macrotis* and *O. roylei* are reported to show local elevation segregation where their geographic range overlap (Dahal et al., 2020; Kawamichi, 1968).

**FIGURE 1:**
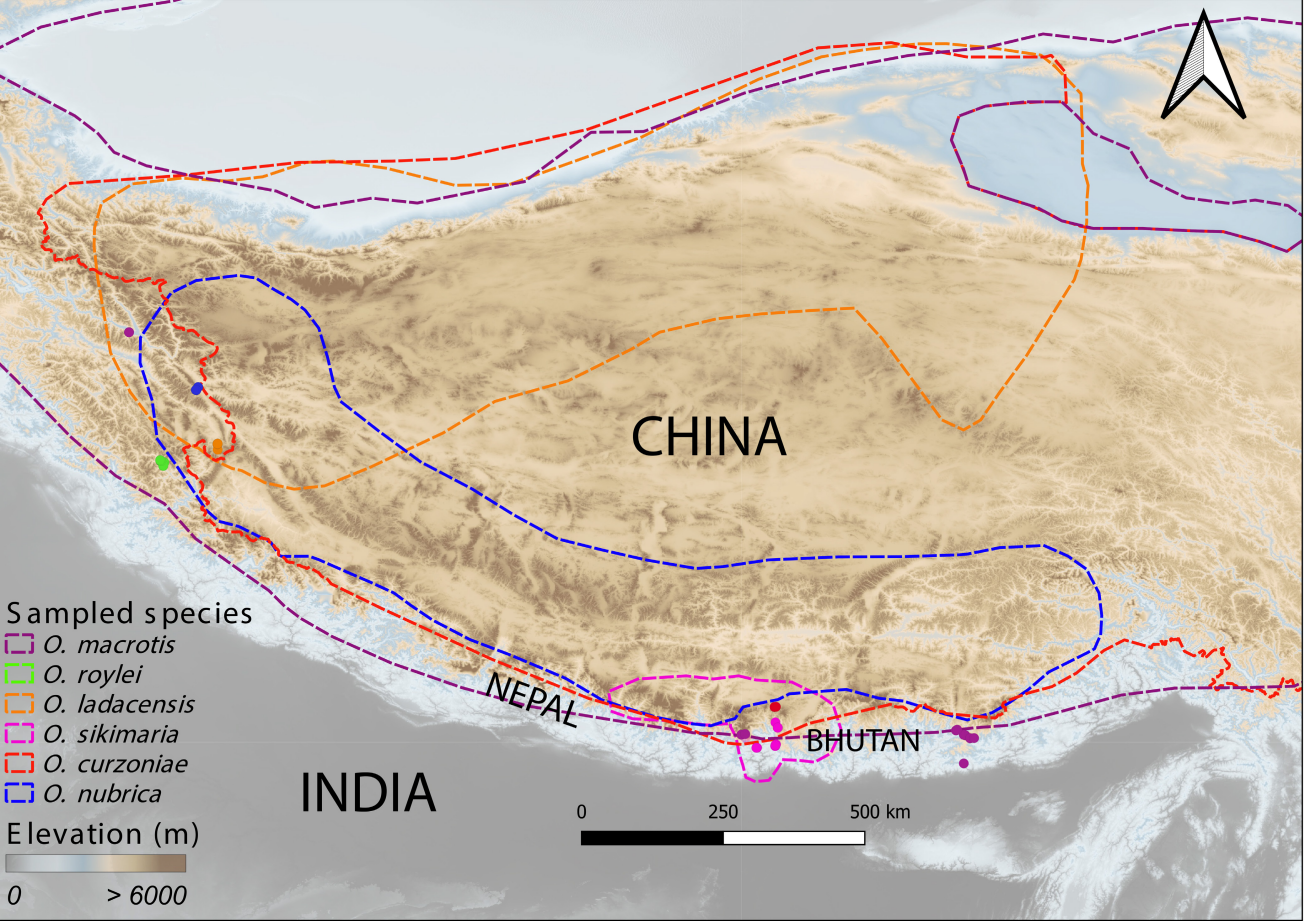
Overall range of all six species of Asian pikas across the Himalayas and Tibetan Plateau used for this study, figure modified from (Dahal et al., 2020). The overall geographic range and sampled locations of the species are color‐coded.

In this study, we have carried out genomic characterization of recently diverged sister species and deeply diverged species from two different subgenera of pikas that are distributed along variable range sizes. We have estimated the genetic diversity (within and between populations) of six species of pikas along their core and southern trailing range edges (Table A1, Figure 1) and tested for evidence of interspecies gene flow. We have utilized these data to examine the patterns of genetic diversity and explored the evidence of gene flow among species distributed along their core and range‐edge habitats in the Himalayas and the neighboring regions.

## 2 METHODS

### 2.1 Study areas and sample collection

**FIGURE 2:**
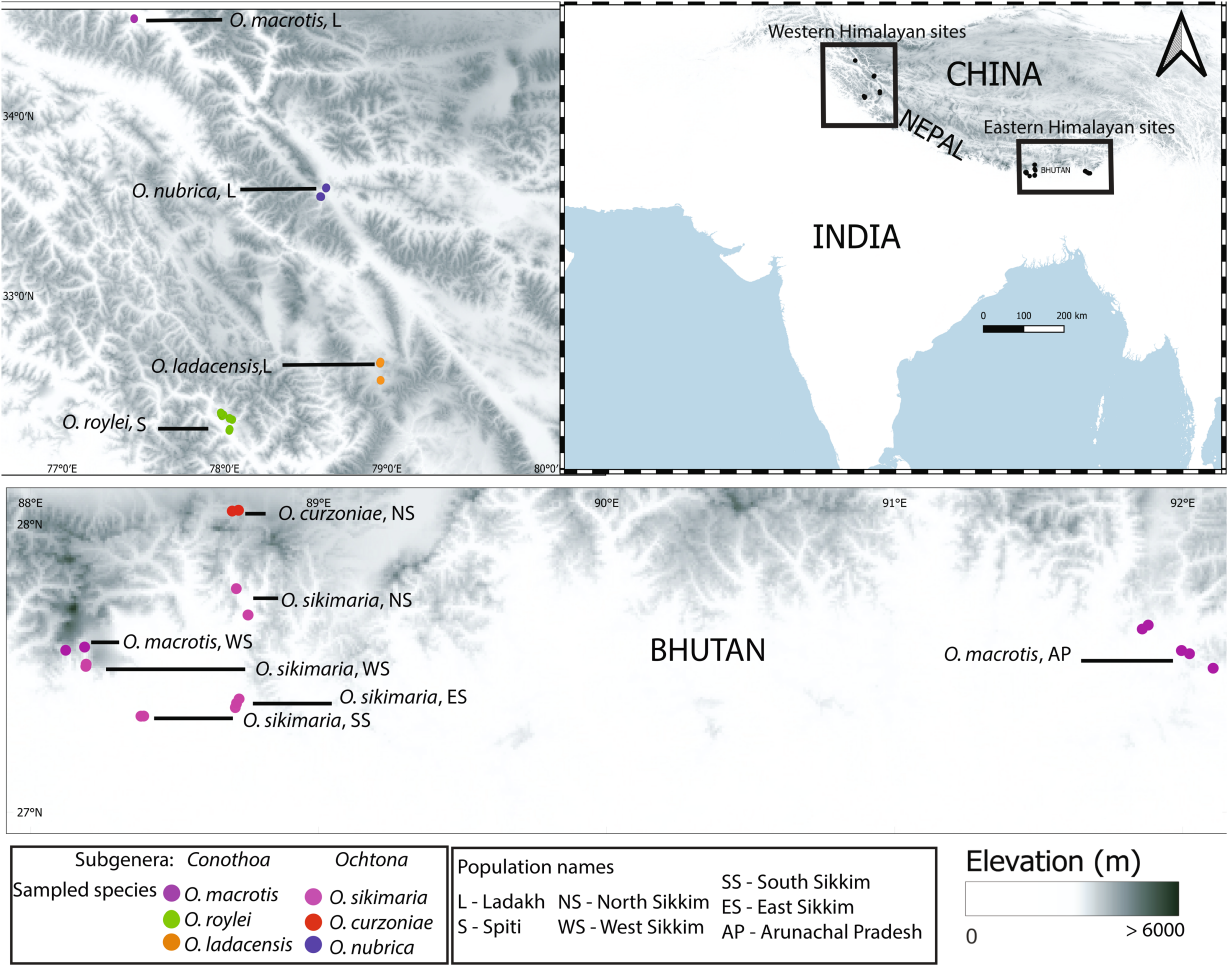
The detailed sampling locations plotted on near 30 m resolution elevation data (source: Shuttle Radar Topography Mission, SRTM). Locations of species sampled in the Eastern and Western Himalayas are marked with boxes (right panel). Zoomed in sampling map of western (top left panel) and eastern (lower panel) Himalayas. The map was made in QGIS version 3.26‐Buenos Aires (http://qgis.org).

Tissue samples were collected during field surveys carried out in the year 2010–2014. Elevational transects were accessed via hiking and traps were set in locations where active pika presence was perceived based on fecal encounter rate and sighting frequencies. Seven sites in the eastern Himalayas and four sites in the western Himalayas were selected for trapping pikas and sampling (Table A1, Figure 2). In each of these locations, trapping was done for a minimum of 3 days, and trapped individuals were immediately released after taking body measurements and ear punch tissues. Tissue samples were collected in 95%–100% ethanol and stored at −20°C until DNA extraction.

### 2.2 Defining “core” and “range‐edge” population

We categorized the population as an edge when it was sampled from either lower or higher elevation limits of the species distribution (Table A1). We also used IUCN red list's range (Figure 1) to categorize population type, for example—*O. nubrica* and *O. ladacensis* populations from Ladakh were grouped as “range‐edge” based on the North‐western and South‐eastern positions of their latitudinal ranges, respectively. For species like *O. roylei* and *O. macrotis* whose overall range has a belt‐like feature, ascertaining range types were mostly based on their elevation limits.

### 2.3 RAD‐ seq library preparation and sequencing

Genomic DNA was extracted from ear punch tissue samples using Qiagen's DNeasy Blood and Tissue kit following the manufacturer's protocol. The double‐digest restriction site‐associated DNA sequencing (ddRADSeq) library was prepared following the protocol described in Peterson et al., 2012. The libraries were sequenced at the Centre for Cellular and Molecular Platforms (C‐CAMP), an initiative supported by the Department of Biotechnology, Govt of India using the Illumina HiSeq platform to generate 100 bp paired‐end reads.

### 2.4 RAD sequences processing

We used STACKS v.2.62 (Catchen et al., 2011) to process the raw sequencing data generated above. Raw sequences were first demultiplexed, barcodes removed, and low‐quality sequences were filtered using “process_radtags.” We then mapped paired end “clean” reads from each sample against the *O. curzoniae* genome downloaded from the NCBI database (Sayers et al., 2022). We aligned short sequence reads to the reference genome using bwa v.0.7.17 (Li & Durbin, 2009) and further converted the alignment into binary format (bam), sorted the alignment, and removed PCR duplicates using Picard v.2.27.5 (http://broadinstitute.github.io/picard/). We then used the “gstacks” program in STACKS to identify SNPs within each population for each locus and then genotyped individuals at each identified SNP (Catchen et al., 2013). We further used the STACKS “populations” program to analyze a population of individual samples and generated aSNP database in VCF format.

The raw variants were then filtered using a variety of parameters as recommended by standard STACKS workflow practices (https://github.com/enormandeau/stacks_workflow). We only selected genotypes with minimum allele coverage = 2, minimum percent of genotype data per population = 40, the maximum number of populations that can fail percent_genotypes = 3, and the minimum number of samples with rare allele = 2. We then created graphs to find samples with high missing data and removed samples with >50% missing genotypes. We further classified SNPs into various categories (singleton, duplicated, diverged, high coverage, low confidence) using the utility scripts provided in STACKS and only kept “singleton” SNPs for downstream analysis. We then estimated pairwise linkage disequilibrium (LD) for each SNP pair using Plink v.1.09 (Chang et al., 2015) and carried out LD‐based pruning of SNPs to keep only one SNP per linked group within the genomic region and make sure there are no spurious correlations among the measured variables. For LD‐based pruning, we defined a genomic window of 50 kb with a 10 bp window step size and prune any SNPs with *r* ^2^ (the measure of LD) > 0.5. Finally, we imputed missing data in the VCF using admixture ancestry relationships (Alexander et al., 2009). The final “clean” SNP dataset consisting of 23,851 variants and 89 samples was used for downstream population genetics analysis.

### 2.5 Estimation of intra‐ and inter‐species genetic diversity

We used vcftools v.0.1.16 (Danecek et al., 2011) to calculate nucleotide diversity (pi) separately for each species/population using the “cleaned” vcf file. We also calculated the inbreeding coefficient (*F*) as a measure of relative heterozygosity for each sample using a method of moments implemented in vcftools. The measure of inter‐species genetic diversity was calculated using the Fixation index (*F*_ST_) (Holsinger & Weir, 2009).

### 2.6 Correlation between genetic and geographic distance

To ascertain if estimates of genetic distance (*F*_ST_) and geographic distance are correlated, we used scatter plots. We used kernel density estimates to demarcate distribution ranges for each population (95% utilization distribution contours). We then extracted the centroid for each of the distribution ranges. The geographic distances were defined as the Euclidean distance between centroids of pairs of population. In addition, we also calculated the nearest distances between pairs of populations using the vertices of the outermost ranges.

### 2.7 Phylogenetic analysis

We examined the genetic relationships among the species by constructing a maximum‐likelihood phylogenetic tree using FastTree (Price et al., 2010) with standard parameters for nucleotide alignments of variable positions in the data set. The local support values for each branch in the tree were calculated using Shimodaira–Hasegawa test implemented in FastTree. Timetree database (Kumar et al., 2017) was used to extract the currently known phylogeny of various species of pikas and their closely related species.

### 2.8 Analysis of population and individual ancestries

We used Admixture v.1.3.0 (Alexander et al., 2009) to estimate individual ancestries from the genome‐wide SNP dataset using a maximum‐likelihood approach. We first explored the optimal number of genetically distinct clusters that best describes the data using a cross‐validation procedure using the *K*‐means method implemented in Admixture. *K* = 7 which had the lowest cross‐validation error was chosen as the optimal fit to describe the genetically distinct clusters among the species being studied. We then ran Admixture to assign a cluster to each individual and calculated the proportion of ancestry to the respective cluster. We further plotted the proportion of ancestry data across all samples.

### 2.9 Exploring evidence of possible gene flow among species

**FIGURE A2:**
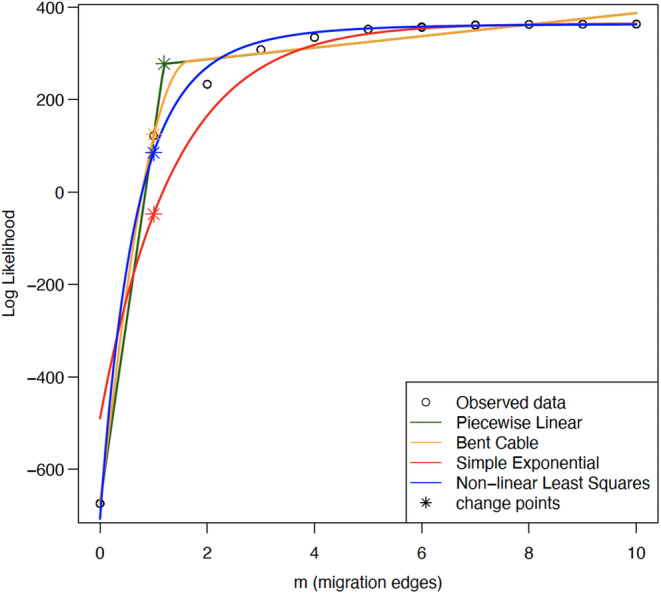
Plot showing changes in log‐likelihood for a possible number of migration events (m). Migration event of either 1 or 2 appears optimum.

We performed three different analyses to examine gene flow among species. First, we used Treemix v.1.13 (Pickrell & Pritchard, 2012) which quantifies the allele frequencies of each SNP to build a maximum‐likelihood phylogenetic tree for all defined populations/species and infers possible admixture events among the branches. We used 500 bootstrap replicates of the SNP data to test for 10 different migration events (10 replicates for each migration event) and choose an optimum number of migration events. The results indicated that a migration event of either 1 or 2 appears optimum (Figure A2). We then carried out two runs of Treemix with migration events 1 and 2 to build a consensus tree and identify possible admixture events. Treemix is a powerful tool for inferring population genetic structure and relationships from genome‐wide genetic data. However, like all statistical models, it is important to assess the adequacy of the model assumptions and examine the residuals to identify any systematic patterns that may indicate problems with the model. We plotted the residuals against the number of migration events inferred by the model (Figure A2). If the residuals exhibit a pattern with respect to the number of migration events, this could suggest that the model is mis‐specified and that additional or different migration event should be included in the model.

Second, we calculated Patterson's *D*‐statistics using ABBA‐BABA tests (Durand et al., 2011; Green et al., 2010) to examine hybridization and possible gene flow among species. In this test, the number of ancestral (“A”) and derived (“B”) alleles are calculated for a four‐taxon comparison that includes an outgroup, predicting patterns termed “ABBA” and “BABA”. Under the condition of incomplete‐lineage sorting without gene flow, the number of SNPs showing pattern “ABBA” and “BABA” should be equally frequent, whereas an excess of either pattern in the genome indicates possible gene flow between taxa that can be calculated using Patterson's *D*‐statistics. We used the Dsuite (Malinsky et al., 2021) for calculating *D* (ABBA/BABA) and f4‐ratio statistics for all trios of species in the dataset. As we did not have an outgroup in this dataset, we used two separate internal outgroups (using a different subgenus) based on the phylogenetic tree generated above, (1) *O. nubrica* (subgenus *Ochotona*) to study the pattern of gene flow between trios of *O. roylei*, *O. macrotis*, and *O. ladacensis* (subgenus *Conothoa*) (2) *O. roylei* (subgenus *Conothoa*) to study the pattern of gene flow between trios of *O. curzoniae*, *O. nubrica*, and *O. sikimaria* (subgenus *Ochotona*). Third, we also inferred pairwise relatedness for each pair of species/population by calculating kinship coefficient using VCFTools.

## 3 RESULTS

### 3.1 Intra‐ and inter‐species genetic diversity

**FIGURE 3:**
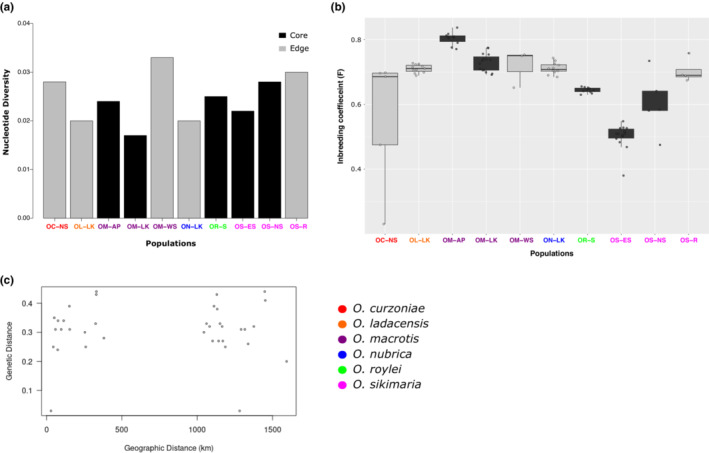
(a) Barplot of nucleotide diversity and, (b) box plot of inbreeding coefficient (*F*) for six populations, color‐coded as core and edge populations (c) scatter plot of pairwise genetic distance (*F*_ST_) plotted against geographic distance (in km).

We used the final filtered SNP dataset consisting of 23,851 variants and 89 samples to calculate genetic diversity (Figure 3a). The average genome‐wide nucleotide diversity was low across all species/populations. We also estimated the inbreeding coefficient (*F*) for each individual as a measure of observed heterozygosity across all samples. The inbreeding coefficient was generally high across all samples, regardless of the population being a “core” or “range‐edge” (Figure 3b). Pairwise‐genetic distance (*F*_ST_) between species ranged from 0.24 to 0.47 and was highest between *O. macrotis* (AP) and *O. sikimaria* (Table A2). Intra‐species *F*_ST_ among different populations ranged from 0.03 to 0.28. The lowest pairwise *F*_ST_ was estimated between *O. macrotis* populations from Ladakh and West Sikkim as 0.03. We also examined if the genetic distance between populations/species correlated with their geographic distribution. However, there was no such correlation (Figure 3c).

### 3.2 Characterization of the population structure

**FIGURE 4:**
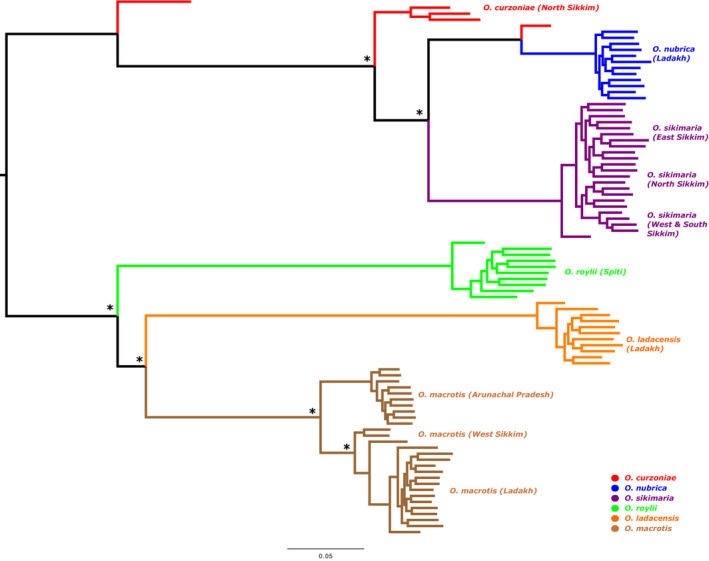
Maximum‐likelihood phylogenetic tree based on 23,851 genome‐wide SNPs and 89 samples from six species of Asian pikas. All nodes having full local support based on the Shimodaira–Hasegawa test (Price et al., 2010) are marked by asterisks.

We first used the TimeTree database (Kumar et al., 2017) to generate an expected phylogenetic tree based on previously published literature (Figure A1). It is expected that *O. nubrica* and *O. curzoniae* being sister species form a phylogenetic cluster and *O. roylei/O. macrotis/O. ladacensis* form a separate cluster. *O. sikimaria* is a newly described species (Dahal et al., 2017) hence is not currently available in the TimeTree database. We further utilized the SNP data to generate an unrooted maximum‐likelihood phylogenetic tree (Figure 4). All 89 samples were clustered into their respective species/populations. As expected, we observed two major phylogenetic clades (1) consisting of *O. macrotis*, *O. ladacensis*, and *O. roylei* (2) consisting of *O. sikimaria*, *O. nubrica*, and *O. curzoniae*. Interestingly, few samples from *O. curzoniae* appear intermediate to both clades.

**FIGURE 5:**
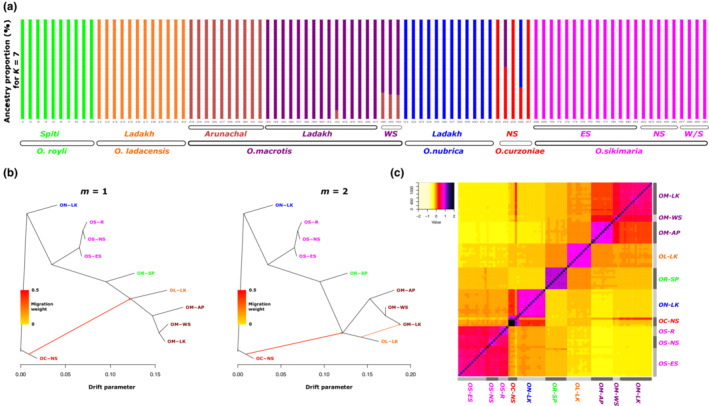
(a) Admixture plot illustrating ancestry among Asian pikas for *K* = 7. Individuals are shown as vertical bars colored in proportion to their estimated ancestry within each cluster (b) Population graphs inferred by TreeMix (Pickrell & Pritchard, 2012) using 23,851 genome‐wide SNPs. Branch lengths are proportional to the evolutionary change (the drift parameter) and terminal nodes are labeled with population codes (see Table A1 for details). TreeMix phylogram is shown with one (left panel) or two migration events (right panel) (c) Heatmap showing relatedness score (Manichaikul et al., 2010) for each pair of individuals calculated using genome‐wide SNP data, Higher values indicate individuals share higher genetic similarities.

We also inferred the population and individual ancestries of all samples using the *K*‐means method using Admixture (Alexander et al., 2009). A cross‐validation procedure indicated *K* = 7 as the optimal number of the genetic cluster (Table A3). The proportion of ancestry for *K* = 7 (Figure 5a) was overall consistent with the phylogenetic tree. We found the *O. macrotis* populations from Ladakh (LK) and West Sikkim (WS) are similar, whereas the Arunachal population (AP) appears genetically distinct. We further calculated pairwise average genetic distance among these three populations of *O. macrotis* (Table 1). The population from AP is 11.24% divergent from LK and 8.78% divergent from WS, indicating its genetic distinctness.

**TABLE 1:** Average pairwise‐genetic distance among three populations of *O. macrotis* (AP: Arunachal Pradesh, WS: West Sikkim, and LK: Ladakh).

	AP	LK	WS
AP	3.66	11.24	8.78
LK	9.87	5.58	7.35
WS	8.78	7.35	3.66

All samples of *O. sikimaria* from different locations in Sikkim as well as *O. roylei* and *O. ladacensis* are genetically distinct (Figure 5a). Some samples of *O. curzoniae* appear to share ancestry with *O. nubrica* and *O. macrotis* samples from Ladakh.

### 3.3 Evidence of gene flow among species

Results from the phylogenetic tree and inference of population and individual ancestries indicated the possibility of gene sharing among some of these species. Hence, we performed three different analyses to examine the possible evidence of gene sharing among different species (1) Treemix (Pickrell & Pritchard, 2012) (2) ABBA‐BABA (Durand et al., 2011; Green et al., 2010), and (3) Genetic relatedness (Manichaikul et al., 2010). We first ran TreeMix with bootstrapping, which helped us to choose an optimum number of migration events in our dataset and create a consensus tree. We plotted the treemix plot with migration events 1 and 2 (Figure 5b). If we consider one migration event, our data indicated gene flow between *O. curzoniae* and *O. ladacensis*. If we consider two migration events, the data indicates (a) the first gene flow between *O. curzoniae* and the base of *O. ladacensis/O. macrotis* split and (b) between Ladakh populations of *O. ladacensis* and *O. macrotis*.

**FIGURE A3:**
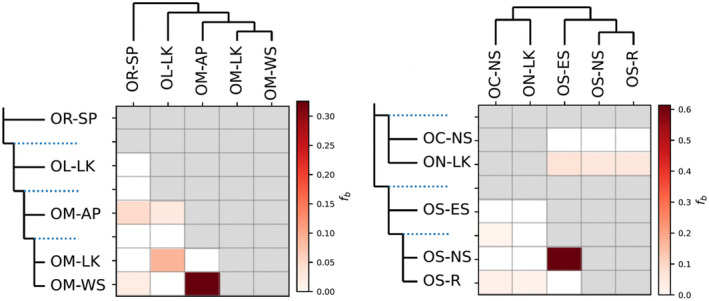
Heatmap summarizing the f‐branch statistics estimated by Dsuite (Malinsky et al., 2021). Darker colors depict increasing evidence for gene flow between lineages. Dotted lines in the phylogeny represent ancestral lineages.

We further examined the pattern of gene flow between species using D and f4‐ratio statistics, based on studying correlations of allele frequencies across populations implemented in Dsuite (Malinsky et al., 2021). As we did not have an outgroup in this dataset, we used two separate internal outgroups based on the phylogenetic tree generated above, (1) *O. nubrica* to study the pattern of gene flow between trios of *O. roylei*, *O. macrotis*, and *O. ladacensis* (2) *O. roylei* to study the pattern of gene flow between trios of *O. curzoniae*, *O. nubrica*, and *O. sikimaria*. For scenario 1, we found evidence of excess allele sharing between populations of *O. macrotis* from AP and WS, but not between AP and LK (Figure A3). We also found evidence of gene flow between Ladakh populations of *O. ladacensis* and *O. macrotis*. For scenario 2, we found evidence of gene flow between populations of *O. sikimaria* and *O. nubrica* (Figure A3).

We also calculated relatedness statistics based on the method of (Manichaikul et al., 2010) for each pair of individuals. The results are consistent with the Treemix and ABBA‐BABA analysis. *O. curzoniae* shows a higher degree of relatedness with *O. macrotis* and *O. nubrica* (Figure 5c). *O. macrotis* populations from Ladakh and West Sikkim are similar, and the Arunachal population appears distinct.

## 4 DISCUSSION

Here, we study six species of Pikas (*Ochotona* spp.) that are distributed across the eastern and western Himalayas and neighboring high‐altitude mountain regions (Figures 1, 2 and Table A1). We utilized a genomic approach using more than 28,000 genome‐wide SNP markers obtained from double digest restriction‐site associated DNA sequencing (ddRAD‐seq) to estimate intra‐ and inter‐species genetic diversity, population structure, phylogenetic history and explore processes that shaped the current genetic diversity of pikas in their core and range‐edge habitat across the Himalayas.

### 4.1 Genetic diversity estimates indicate population decline in Asian pikas

The nucleotide diversity was consistently low across all species (Figure 3a) whereas the inbreeding coefficient was high (>0.5) (Figure 3b). These two estimates indicated that all six species of pikas in this study have low genetic diversity, regardless of the populations/species residing in their core and/or range‐edge habitat. The core populations included in the current study include species like *O. macrotis*, *O. roylei*, and *O. nubrica*, which have a “belt‐like” habitat distribution, with probably no core population, which could reflect in their overall high inbreeding coefficient and low nucleotide diversity. Low genetic diversity has been a common feature in one of the highly studied species, American pikas (*O. princeps*) which has been associated with elevation‐specific population decline and restricted dispersal (Klingler et al., 2021; Robson et al., 2016). A previous study in *O. curzoniae* using 10 microsatellite markers also identified a high inbreeding coefficient in this species (Zhang et al., 2017). Our results of low genetic diversity in these six species of Asian pikas also indicate historically small population size and/or ongoing population decline in pikas. In addition, the Himalayas are the southern trailing range edge for most of these species (Table A1), especially *O. curzoniae* and *O. ladacensis* (Dahal et al., 2020) which may also contribute to low population size and thereby, low nucleotide diversity.

### 4.2 Is the genetic distance explained by geographic distance?

There is often a correlation between genetic and geographic distance and low genetic diversity (Eckert et al., 2008). Populations that are geographically isolated and have limited gene flow with other populations are more likely to have low genetic diversity (Malécot, 1948). This is because genetic diversity is typically maintained through the mixing of genetic material between populations, which can introduce new genetic variations and prevent the accumulation of deleterious mutations (Lande & Barrowclough, 1987). When populations are isolated geographically, they may experience genetic drift, which is the random fluctuation of allele frequencies due to chance events. Genetic drift can lead to the loss of genetic variation over time, particularly in small populations (Lacy, 1997). Additionally, populations that have gone through a bottleneck event, where the population size has dramatically decreased, are also more likely to have low genetic diversity due to the random loss of genetic variation (van Straalen & Timmermans, 2002).

Overall, we did not see a correlation between pairwise genetic differentiation (*F*_ST_) and geographic distance (Figure 3c) among populations of six species of pikas in our study.

Inter‐species pairwise genetic differentiation (*F*_ST_) was generally high for each species pair (Table A2). The intra‐species pairwise genetic differentiation of *O. macrotis*, the widest‐ranging species for which we had samples from three different latitudinal regions did not correspond to the geographic distance. For example, the population of *O. macrotis* from Sikkim showed high genetic differentiation (*F*_ST_ = 0.28) with the population from Arunachal Pradesh (both populations from eastern Himalayas, less geographic distance) whereas very low genetic differentiation with the population from Ladakh (populations from western Himalayas, high geographic distance) (*F*_ST_ = 0.03). Our results indicated that geographic distance cannot fully explain the genetic distance between populations/species of six species of Pikas in the Himalayas.

### 4.3 Evidence of gene flow between species

Our results indicated that genetic diversity was relatively consistent among all species, regardless of a population we defined as residing in “core” or “range‐edge” habitat, possibly indicating case of gene flow among these species. It is known that gene flow can introduce new genetic variation into a population, which can help to maintain genetic diversity and increase the adaptive potential of the population (genetic rescue hypothesis) (Tallmon et al., 2004). Cases of interspecific hybridization and gene flow among three species of subgenus *Ochotona* has been recently validated with genomics data (Lissovsky et al., 2019). We examined the patterns of gene flow among the six species of Asian pikas to test the genetic rescue hypothesis using three different approaches. First, we examined the phylogenetic tree based on SNP data (Figure 4), which was consistent with previously known phylogeny. *Ochotona macrotis*, *O. ladacensis*, and *O. roylei* were grouped into one cluster as these species are from the same subgenus (*Conothoa*). Similarly, *O. sikimaria*, *O. nubrica*, and *O. curzoniae* were grouped into another cluster as these species are from different subgenus (*Ochotona*). Interestingly, few samples from *O. curzoniae* appear intermediate to both clades which is a possible indication that this species is possibly being exposed to gene flow. A recently published study has identified strong evidence of gene flow from *O. curzoniae* in their “core” habitat to ecologically distinct range‐edge species (Ge et al., 2023).

We also examined the inference of population ancestry (Figure 5a) and the pattern of admixture among populations/species. Some *O. curzoniae* individuals appeared to have mixed ancestry which was consistent with their relatively intermediate position in the phylogenetic tree. Population from the range‐edge habitat in *O. macrotis* shared most ancestry with the population of the same species from the core habitat, however, this “range‐edge” population from west Sikkim (eastern Himalayas) was genetically similar to a geographically distant core population from Ladakh (western Himalayas) in comparison to another core population from a geographically closer core population from Arunachal Pradesh. These results are consistent with patterns of genetic divergence (*F*_ST_) we have identified among these three populations of *O. macrotis* (Table A2). The genetic similarity of two geographically distant populations of *O. macrotis* is contrary to the theory of isolation by distance (IBD), which states that genetic similarity decreases among populations as the geographic distance between them increases (Wright, 1938, 1940). As *O. macrotis* has a wider distribution range across the eastern and western Himalayas (Figure 1), this observed genetic similarity among geographically distant populations possibly indicates patterns of historic gene flow in their ancestral core and range‐edge populations. Our results indicated that the *O. macrotis* population from Arunachal Pradesh is genetically distinct. This result supports the previous classification of this population as a separate subspecies of *O. macrotis* (Lissovsky et al., 2017).

Treemix analysis indicated possible gene flow between *O. curzoniae* and *O. ladacensis*. Taxonomically, these species are non‐sibling species from different subgenera, so sharing of genes between these two species was unexpected. However, the distribution range of these two species overlaps at their range edge (Dahal et al., 2020) and shares a similar ecological niche (Table A1). Hence, these species have a possibility of sharing genes as indicated by our results. Treemix analysis also identified evidence of gene flow among the range‐edge population of *O. ladacensis* and the core population of *O. macrotis* in Ladakh (western Himalayas). We expect a similar case of gene flow in these two species that overlap in their distribution range in the western Himalayas. Consistent with the results of Treemix, the evidence of gene flow among populations of *O. ladacensis* and *O. macrotis* in Ladakh populations is also independently identified in ABBA‐BABA analysis (Figure A3). ABBA‐BBA also identified evidence of gene flow between populations of *O. sikimaria* and *O. nubrica*. The case of gene flow between *O. nubrica* and *O. sikimaria* is consistent with previously hypothesized cases of interspecific hybridization and introgression among these species (Lissovsky et al., 2019). Finally, the estimates of pairwise relatedness scores among species/populations were also consistent with the results of Treemix and ABBA‐BABA analysis (Figure 5c). Our results support the genetic rescue hypothesis that is possibly maintaining genetic diversity in geographically restricted pika species like O. *ladacensis O. nubrica* and *O. sikimaria* which is vulnerable to historic population decline and inbreeding depression. Inter‐species gene flow between species from divergent sub‐genera was not reported in earlier studies in pikas. However, our result is consistent with a recent study (Ge et al., 2023) that has identified that *O. curzoniae*, with a wide central range in the Tibetan Plateau, shows gene flow with other species in the peripheral Himalayan range.

Genetic diversity is a major contributor to the evolutionary viability of natural populations and has a key implication in species conservation (Frankham, 1995). Low genetic diversity indicates that the effective population size of Asian pikas is possibly declining. These results are consistent with species distribution models predicted for future habitat loss in certain species of pikas e.g. *O. roylei* (Bhattacharyya et al., 2019). Although Asian Pikas currently have “Least Concern” status on IUCN red list (IUCN, 2017), our results indicate the immediate need for effective management and monitoring practices for pikas.

While the study highlights the implication of inter‐species gene flow as a key biological process associated with pika evolution, the implication of gene flow, whether negative or positive in the overall adaptation and fitness of the species still needs to be investigated. The knowledge about the direction of gene flow and functional attributes of introgressed regions in the genome is key to drawing any conclusion on the conservation implication of ongoing inter‐species gene flow in pikas. However, high‐quality reference genomes of these species and population‐scale whole‐genome sequencing will be required in the future to better characterize genomic signatures of species hybridization and gene flow.

## AUTHOR CONTRIBUTIONS

**Nishma Dahal:** Conceptualization (equal); formal analysis (equal); investigation (equal); methodology (equal); writing – original draft (equal); writing – review and editing (equal). **Melia G Romine:** Investigation (supporting); methodology (supporting); writing – review and editing (supporting). **Sunita Khatiwara:** Methodology (supporting); writing – review and editing (supporting). **Uma Ramakrishnan:** Conceptualization (equal); investigation (equal); writing – review and editing (equal). **Sangeet Lamichhaney:** Conceptualization (equal); investigation (equal); methodology (lead); writing – original draft (lead); writing – review and editing (lead).

## Data Availability

All genomic data generated in this study are available via NCBI BioProject, accession number PRJNA893189 (https://www.ncbi.nlm.nih.gov/bioproject/PRJNA893189).

## APPENDIX 1

**TABLE A1:** Sampling location and brief description of habitat and ecology of species studied.

Species pop code (number of samples)	Sampled location/range	Sampled elevation (in m)	IUCN red list elevation range (in m)	Subgenera	Sampled habitat type	Species' ecology	Core or range edge
*O. curzoniae* OC‐NS (5)	North Sikkim, Eastern Himalaya	5060–5067	3000–5000	*Ochotona*	Open desert‐steppe	Burrowing pika, makes complex burrows; social	Upper elevation limit and southern trailing edge
*O. ladacensis* OL‐L (11)	Hanle, Ladakh, Western Himalaya	4840–4888	4200–5400	*Conothoa*	Open desert‐steppe	Burrowing pika, social	Core elevation but southern range edge
*O. macrotis* OM‐AP (10)	Tawang, Arunachal Pradesh, Eastern Himalaya	3311–4213	2300–6400	*Conothoa*	Big rocky patches	Talus dweller, asocial	Core elevation and range
*O. macrotis* OM‐L (15)	Hunder, Ladakh, Western Himalaya	3205–3212	2300–6400	*Conothoa*	Rocky patches near stream	Talus dweller, asocial	Core elevation and range
*O. macrotis* OM‐WS (3)	Kanchenjunga National Park, West Sikkim, Eastern Himalaya	4559–4754	2300–6400	*Conothoa*	Big rocky patches near lake	Talus dweller, asocial	Towards upper elevation limits but core range
*O. nubrica* ON‐L (12)	Chushul, Ladakh, Western Himalaya	4330–4403	3000–4500	*Ochotona*	Shrub steppe marshes, dominated by *Caragana* sp.	Burrowing pika (shallow burrower), shy	Towards upper elevation limits and north‐western range edge
*O. roylei* OR‐S (10)	Spiti, Himachal Pradesh, Western Himalaya	3649–4997	2400–5200	*Conothoa*	Rocky patches	Talus dweller, asocial	Core range
*O. sikimaria* OS‐ES (14)	Kyongnosla Alpine Sanctuary, East Sikkim, Eastern Himalaya	3096–3666	Not assessed	*Ochotona*	Sub‐alpine Rhododendron forest	Burrows in open habitat with many entrances; burrows in farmlands are less complex; social	Core range
*O. sikimaria* OS‐NS (5)	Lachung, North Sikkim, Eastern Himalaya	2961–3509	Not assessed	*Ochotona*	Sub‐alpine forest: Farmland and Rhododendron forests.	Burrows in open habitat with many entrances; burrows in farmlands are less complex; social	Core range
*O. sikimaria* OS‐SS (4)	Maenam Wildlife Sanctuary, South Sikkim & Kanchenjunga National Park, West Sikkim, Western Himalaya	3233–4203	Not assessed	*Ochotona*	Sub‐alpine forest dominated with shrubs of R*hododendron* sp. and *Gaultheria* sp.	Burrows in open habitat with many entrances; burrows in farmlands are less complex; social	Towards upper elevation range limits

**TABLE A2:** Pairwise *F*_ST_ between species/populations.

	*O. curzoniae*‐North Sikkim	*O. ladacensis*‐Ladakh	*O. macrotis*, Arunachal Pradesh	*O. macrotis*‐Ladakh	*O. macrotis*, West Sikkim	*O. nubrica*‐Ladakh	*O. roylei*, Spiti	*O. sikimaria*, East Sikkim	*O. sikimaria*, North Sikkim	*O. sikimaria*, south & West Sikkim
*O. curzoniae*‐North Sikkim	0	0.33	0.44	0.31	0.34	0.27	0.38	0.24	0.25	0.26
*O. ladacensis*‐Ladakh	0.33	0	0.32	0.25	0.3	0.34	0.31	0.27	0.32	0.34
*O. macrotis*, Arunachal Pradesh	0.44	0.32	0	0.2	0.28	0.44	0.41	0.33	0.43	0.47
O. macrotis‐Ladakh	0.31	0.25	0.2	0	0.03	0.31	0.3	0.26	0.31	0.33
*O. macrotis*, West Sikkim	0.34	0.3	0.28	0.03	0	0.43	0.39	0.31	0.35	0.39
*O. nubrica*‐Ladakh	0.27	0.34	0.44	0.31	0.43	0	0.39	0.25	0.32	0.35
*O. roylei*, Spiti	0.38	0.31	0.41	0.3	0.39	0.39	0	0.27	0.33	0.36
*O. sikimaria*, East Sikkim	0.24	0.27	0.33	0.26	0.31	0.25	0.27	0	0.03	0.05
*O. sikimaria*, North Sikkim	0.25	0.32	0.43	0.31	0.35	0.32	0.33	0.03	0	0.02
*O. sikimaria*, South & West Sikkim	0.26	0.34	0.47	0.33	0.39	0.35	0.36	0.05	0.02	0

**TABLE A3:** Cross‐validation error (CV) for *k* = 1–11 for admixture analysis. The lowest error *K* = 7) indicated the optimal number of genetic clusters.

CV error (*K* = 1): 0.85100
CV error (*K* = 2): 0.48564
CV error (*K* = 3): 0.36538
CV error (*K* = 4): 0.28738
CV error (*K* = 5): 0.22379
CV error (*K* = 6): 0.21977
CV error (*K* = 7): 0.19834
CV error (*K* = 8): 0.22517
CV error (*K* = 9): 0.21435
CV error (*K* = 10): 0.26099
CV error (*K* = 11): 0.27969
